# Targeting Inflammasome Activation in COVID-19: Delivery of RNA Interference-Based Therapeutic Molecules

**DOI:** 10.3390/biomedicines9121823

**Published:** 2021-12-03

**Authors:** Lealem Gedefaw, Sami Ullah, Thomas M. H. Lee, Shea Ping Yip, Chien-Ling Huang

**Affiliations:** 1 Department of Health Technology and Informatics, The Hong Kong Polytechnic University, Hong Kong, China; lealem.bimerew@connect.polyu.hk (L.G.); samikhan.ullah@polyu.edu.hk (S.U.); 2 Department of Biomedical Engineering, The Hong Kong Polytechnic University, Hong Kong, China; ming-hung.lee@polyu.edu.hk; 3 Research Institute for Future Food, The Hong Kong Polytechnic University, Hong Kong, China

**Keywords:** inflammasome, RNA interference, COVID-19, non-coding RNAs, molecular targets

## Abstract

Mortality and morbidity associated with COVID-19 continue to be significantly high worldwide, owing to the absence of effective treatment strategies. The emergence of different variants of SARS-CoV-2 is also a considerable source of concern and has led to challenges in the development of better prevention and treatment strategies, including vaccines. Immune dysregulation due to pro-inflammatory mediators has worsened the situation in COVID-19 patients. Inflammasomes play a critical role in modulating pro-inflammatory cytokines in the pathogenesis of COVID-19 and their activation is associated with poor clinical outcomes. Numerous preclinical and clinical trials for COVID-19 treatment using different approaches are currently underway. Targeting different inflammasomes to reduce the cytokine storm, and its associated complications, in COVID-19 patients is a new area of research. Non-coding RNAs, targeting inflammasome activation, may serve as an effective treatment strategy. However, the efficacy of these therapeutic agents is highly dependent on the delivery system. MicroRNAs and long non-coding RNAs, in conjunction with an efficient delivery vehicle, present a potential strategy for regulating NLRP3 activity through various RNA interference (RNAi) mechanisms. In this regard, the use of nanomaterials and other vehicle types for the delivery of RNAi-based therapeutic molecules for COVID-19 may serve as a novel approach for enhancing drug efficacy. The present review briefly summarizes immune dysregulation and its consequences, the roles of different non-coding RNAs in regulating the NLRP3 inflammasome, distinct types of vectors for their delivery, and potential therapeutic targets of microRNA for treatment of COVID-19.

## 1. Introduction

Severe acute respiratory syndrome coronavirus 2 (SARS-CoV-2) is the causative agent of the coronavirus disease, 2019 (COVID-19), which first originated in the Chinese city of Wuhan and has since claimed the lives of over 5.1 million individuals worldwide [1]. The clinical presentation of the disease varies from less severe symptoms, such as mild pneumonia, to critical cases, including acute respiratory distress syndrome (ARDS). In patients with severe symptoms, immune dysregulation, characterized by a hyper-inflammatory response, is common, potentially leading to organ failure and death [2,3]. The inflammasome is a multi-protein complex that cleaves and activates pro-inflammatory elements, such as interleukin-1beta (IL-1β) and IL-18, and promotes a hyper-inflammatory response. The inflammasome complex typically has three constituent protein subunits: (1) a sensor protein, such as nucleotide oligomerization domain-like (NOD-like) receptors (NLRs) or absent in melanoma 2 (AIM2)-like receptors; (2) an adapter protein known as an apoptosis-associated speck-like protein with a caspase recruitment domain (ASC); and (3) the pro-caspase 1 zymogen. Inflammasomes are also involved in inflammation-mediated cell death (pyroptosis) through cleavage of the gasdermin D (GSDMD) protein [4,5].

SARS-CoV-2 consists of several structural proteins, such as nucleocapsid (N), spike (S), membrane (M), and envelope (E) proteins. Viral entry into the host is facilitated by the binding of a viral S protein to angiotensin-converting enzyme 2 (ACE2) receptors on lung cells. Both structural and non-structural SARS-CoV-2 proteins have been associated with inflammasome activation and accompanying inflammation. Viroporins are viral proteins capable of activating inflammasomes, which can modify the cell membrane for the entry of viruses into the host cells. After endocytosis, the viral E protein polymerizes and facilitates the release of calcium ion (Ca^2+^) from the Golgi apparatus, consequently activating the inflammasome. Additionally, non-structural accessory proteins of SARS-CoV-2, including open reading frame 3a (orf3a) and orf8b proteins, trigger inflammasome activation and pyroptosis by potentially causing ionic imbalance along the cell membrane, mitochondrial dysfunction producing reactive oxygen species (ROS), and activating intracellular stress pathways [4,6,7,8].

Several studies have recently demonstrated the functional crosstalk between non-coding RNAs (ncRNAs) and the NLRP3 inflammasome to regulate inflammatory responses during specific pathophysiological conditions [9,10]; NLRP3 stands for the protein NLR family pyrin domain containing 3. Among the short ncRNAs, the endogenous single-stranded microRNAs (miRNAs), 18–24 nucleotides in length, play a critical role in disease progression, based on their potential to regulate gene expression at different levels [11]. These miRNAs target several genes and other components [12] and can positively regulate NLRP3 inflammasome activation by promoting the assembly of NLRP3 components, neutralizing the inhibitory effects of certain miRNAs, and enhancing the expression of pro-inflammatory genes [13,14,15]. On the other hand, ncRNA transcripts can also negatively influence NLRP3 activity by inhibiting the expression of NLRP3 components and suppressing the thioredoxin-interacting protein (TXNIP), which can trigger NLRP3 activation or prevent excessive inflammation by regulating downstream cytokine expression [16,17,18].

Based on the regulation of NLRP3 inflammasome by ncRNAs, novel therapeutic strategies against viral infections are in development. However, RNA molecules are naturally unstable, and these strategies are hampered by the lack of effective delivery systems [18]. Certain transcript modifications are also required to avoid the host immune system and non-specific interactions [19]. Recently, the introduction of synthetic delivery vehicles has improved the therapeutic efficacy of RNA-based drugs [20]. Cyclodextrin polymer-based nanoparticles (NPs) [21], lipid NPs (liposomes) [22,23], polymers, aptamers, peptides, antibodies, and other small molecules [24] are promising delivering molecules. Recently, exosomes released from mesenchymal stem cells (MSCs), clustered regularly interspaced short palindromic repeats associated proteins (CRISPR-Cas) technologies, and messenger ribonucleic acid (mRNA) vaccines have also emerged as promising tools for delivering different RNA molecules [25,26,27] to combat the current pandemic.

The role of NLRP3 in different pathophysiological conditions has been widely investigated. Although the defensive role of NLRP3 is well documented, hyper-inflammatory responses, triggered by its downstream effector molecules, could lead to a higher risk of adverse events, such as autoimmune and other metabolic diseases [9]. Thus, inflammasome activity needs to be regulated in both nuclear and cytoplasmic compartments [9].

Despite the fact that dysregulation of pro-inflammatory cytokines may play a role in lung injury and SARS-CoV-2 pathogenesis, the underlying molecular pathways and treatment methods have yet to be discovered. In this review, we shall briefly discuss current updates on immunological dysregulation caused by COVID-19 inflammasome activation, the regulatory effects of ncRNAs on inflammasomes, and delivery options for COVID-19 RNA-based therapies.

## 2. Immune Dysregulation in COVID-19

Inflammation is an obligatory part of an effective immune response, without which, successful elimination of an infectious agent is difficult. The immune response to viral infection is usually investigated at the cellular level, following viral entry into the target cell, which subsequently mediates immune cell recruitment, removes pathogens, and eventually culminates in tissue repair and returns to equilibrium [28]. SARS-CoV-2 first binds to the secondary pneumocytes (alveolar type 2 cells) in the lungs and damages the infected cells and other related cells. This induces disruption of both epithelial and endothelial cells in the lungs, together with an alveolar inflammatory cell infiltration, resulting in pro-inflammatory cytokines being released early. The pro-inflammatory cytokines released by the damaged cells induce excessive and prolonged cellular activation, mainly via macrophages [29]. In COVID-19 patients, a significant number of abnormal macrophages were found in alveolar cavities [30], which could lead to macrophage activation syndrome [31]. The occurrence of macrophage activation syndrome and poor antigen presentation, caused by interleukin 6 (IL-6), are predisposing factors for severe respiratory failure [31]. In addition to type 2 pneumocytes, SARS-CoV-2 also directly infects the pulmonary endothelial cells and induces vascular injury. Both in vitro and in vivo experiments proved that SARS-CoV-2 induced hyper-inflammation through causing vascular damage, and this contributed to multi-organ failure [32,33].

Neutrophils are the most common white blood cells and serve as the first line of defense against infections. Chemical signals stimulate neutrophil chemotaxis and recruitment to the site of infection, where neutrophils gather together and cause the release of more pro-inflammatory mediators, including free radicals [29,34]. Qin et al. have demonstrated that, in COVID-19 patients, immune dysregulation is characterized by elevated levels of infection-related biomarkers and inflammatory cytokines. Several inflammatory cytokines were elevated in severe cases when compared with non-severe cases, including IL-1β, IL-6, IL-8, IL-10, granulocyte colony-stimulating factor, granulocyte monocyte colony-stimulating factor, interferon-gamma, interferon-gamma inducible protein 10, monocyte chemoattractant protein-1, macrophage inflammatory proteins alpha, and tumor necrosis factor-alpha [34,35]. Low expression of human leukocyte antigen–DR isotype and lymphopenia are defined by IL-6-mediated immune dysregulation in severe COVID-19, which is associated with continuous cytokine production and hyper-inflammation [31].

Immune dysregulation with a marked reduction in immune cells is associated with respiratory failure in COVID-19 patients [31]. This dysregulated immune response is excessive in critically ill COVID-19 patients, resulting in a systemic “cytokine storm” that precipitates the advent of a systemic inflammatory response syndrome, and leads to hypercoagulopathy, organ failure, and death [36,37]. The cytokine storm, regularly observed in COVID-19 patients, is also characterized by inflammasome development, as demonstrated in numerous clinical studies [38].

In leukocytes, an elevated inflammatory expression is found in patients who have died from COVID-19. In peripheral blood mononuclear cells, active NLRP3 inflammasome was observed and was associated with disease severity in COVID-19 patients [3]. In COVID-19 pneumonia, the occurrence of fatal NLRP3 inflammasome aggregates in the lungs indicated a possible molecular interaction between viral infection and cytokine storm formation [39]. The innate NLRP3 inflammasome immune signal receptor is involved in the cleavage and activation of pro-inflammatory cytokines that lead to the activation of inflammatory molecules such as IL-1β and active caspase 1. These pro-inflammatory cytokines cause pyroptosis (a rapid, pro-inflammatory cell death process) and inflammation of the tissues [40]. A better understanding of the mechanism of inflammasome activation and its interaction with innate immune pathways could lead to the development of new antiviral drugs promoting innate immune response.

## 3. Inflammasome Activation in Coronavirus Infections

Our bodies are constantly exposed to viral infections, and the immune system works efficiently to protect us against disease. Two general forms of the immune response (innate and adaptive) are activated following a viral infection. The expression of pro-inflammatory cytokines is one of the key responses of the innate immune system to viral particles. The adaptive immune response becomes more robust if the condition persists beyond the first few viral replication rounds [41,42].

Primary cells of innate effectors, such as macrophages, are actively involved in the engulfment of viral particles and the release of signals that activate other immune cells to protect against viral entry via pattern recognition receptors (PRRs). Upon infection with microbes such as bacteria, viruses, fungi, and protozoa, PRRs serve as innate immune sensors for pathogen-associated molecular patterns (PAMPs). Viral PAMPs comprise double-stranded RNA, single-stranded RNA (ssRNA), and cytoplasmic genomic DNA. Damage-associated molecular patterns (DAMPs), such as host DNA/RNA, adenosine triphosphate, uric acid, or ROS, are also recognized by these receptors [43]. Other host cell receptors that recognize viruses include NLRs, AIM2s, and RIG-1-like receptors, which form complexes of inflammasomes [44].

Inflammasome surfaces usually attract the inactive pro-caspase 1 zymogen. Among the NLRs, NLRP3, also known as cryopyrin, is the most extensively investigated sensor molecule. NLRP3 is encoded by the *NLRP3* gene on chromosome 1. Activation of the NLRP3 inflammasome is stringently regulated and involves a two-phase process (priming and activation) to prevent an excessive inflammatory response. NLRP3 is involved in the host immune response to several RNA virus types, including Ebola virus [45], influenza A virus (IAV) [46], hepatitis C virus [47], and human immunodeficiency virus [48], as well as DNA viruses including adenovirus [49], varicella-zoster virus [50], herpes virus [51], and coronavirus [52].

**Figure 1:**
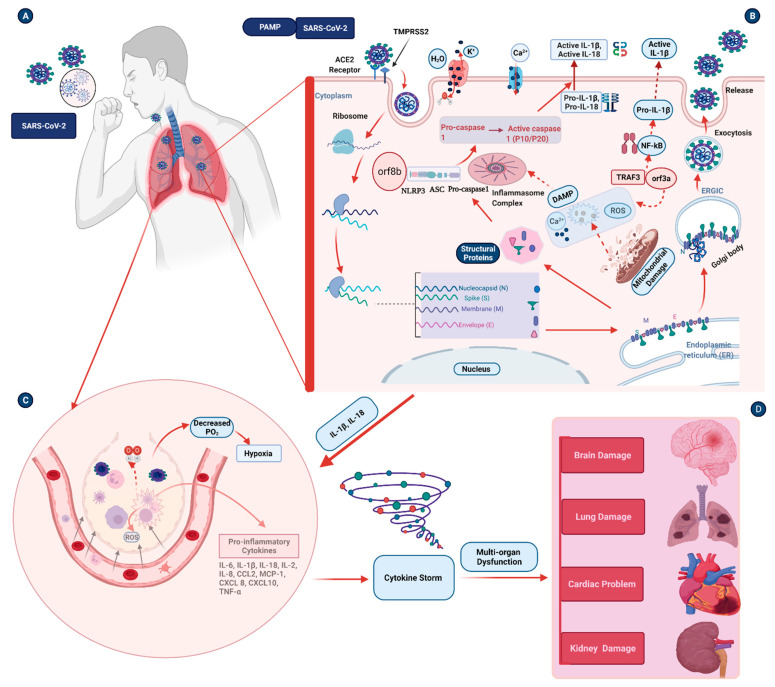
Mechanisms of inflammasome activation in SARS-CoV-2 infection. (**A**,**B**) PAMPs, such as SARS-CoV-2, are inhaled into the lungs. SARS-CoV-2 enters the lung cells through ACE2 receptors. The virus uses the machinery of host cells for replication and synthesis of its proteins during the replication process in the host cells. These structural (S, N, M, and E proteins) and non-structural proteins (orf8b and orf3a) of SARS-CoV-2 can influence ion exchange and damage intracellular organelles. This results in the release of Ca^2+^ and ROS. Via TRAF3, orf3a upregulates NF-kB, and the NF-kB can induce the release of IL-1β. This process will lead to upregulation and activation of inflammasome genes. The inflammasome components are assembled and in turn activate the pro-caspase 1. The active caspase 1 activates pro-IL-1β and pro-IL-18 into active IL-1β and active IL-18. These pro-inflammatory cytokines induce inflammation. (**C**) Invasion of the lung cells by SARS-CoV-2 results in tissue damage, macrophage activation, and hypoxia. Other immune cells will also migrate into the lung tissues and cause excessive immune activation and release of pro-inflammatory cytokines. (**D**) Cytokine storms caused by excessive immune activation and release of pro-inflammatory cytokines result in multi-organ damage, including brain damage, lung damage, cardiac problems, and kidney damage. ACE2—angiotensin-converting enzyme 2; ASC—a speck-like protein containing a caspase recruitment domain CARD; Ca^2+^—calcium ion; CCL2—chemokine (C-C motif) ligand 2; CXCL8—chemokine (C-X-C motif) ligand 8; CXCL10—C-X-C motif chemokine ligand 10; DAMPs—damage-associated molecular patterns; ERGIC—endoplasmic-reticulum–golgi intermediate compartment; H_2_O—water; IL-1β—interleukin 1 beta; IL-2—interleukin 2; IL-6—interleukin 6; IL-18—interleukin 18; K^+^—potassium; NF-kB—nuclear factor kappa light chain enhancer of activated B cells; NLRP3—nucleotide-binding oligomerization domain, leucine-rich repeat, and pyrin domain-containing 3 protein; orf3a—open reading frame 3a; orf8—open reading frame 8; PAMPs—pathogen-associated molecular patterns; PO_2_—partial pressure of oxygen; ROS—reactive oxygen specious; TMPRSS2—type II transmembrane serine protease2; TNF α—tumor necrosis factor α; TRAF3—TNF receptor-associated factor 3. The solid arrow indicates the direction of effect; the dotted arrow indicates alternative mechanisms.

Coronaviruses are a diverse group of viruses that often cause mild–moderate upper respiratory tract infections. Various studies suggest that structural and accessory proteins of coronaviruses induce inflammasome activation. The structural proteins of coronaviruses are reported to improve the control of the inflammasome pathway. Activation of the NLRP3 inflammasome in alveolar macrophages by the coronavirus family appears to be mediated through various mechanisms. For instance, the E protein is richly expressed within infected cells during viral replication, and a small proportion is incorporated into the envelope of virions. Most proteins are located at intracellular trafficking sites, such as the Golgi apparatus, where they participate in the assembly and budding of virus and regulation of the Ca^2+^ ion channel. The E protein ion channel enhances NLRP3 inflammasome activation, leading to IL-1β overproduction [53]. Similarly, the inflammasome proteins and pro-inflammatory cytokines can also be primed and activated by the SARS-CoV-2 S protein [54] and N protein [55]. Orf3a encodes the SARS-CoV-2 viroporin, which triggers the NLRP3 inflammasome. Orf3a interacts with TNF receptor-associated factor 3 (TRAF3) and promotes TRAF3-dependent ubiquitination of ASC, which activates the protein complex nuclear factor kappa light chain enhancer of activated B cells (NF-kB) and the NLRP3 inflammasome [7]. Orf3a requires potassium (K^+^) efflux and oligomerization between NIMA-related kinases (NEK7) and NLRP3 to induce IL-1β expression via NF-kB [52] (Figure 1). Impaired inflammasome activation could lead to increased immune cell death [56]. On the other hand, the absence of inflammatory components of NLRP3 and GSDMD during infection with coronavirus leads to an initial reduction in cell death, accompanied by a robust increase in the incidence of inflammatory cell death, mediated by two enzymes, namely caspase 8 and receptor-interacting serine/threonine-protein kinase 3 [56].

The NLRP3 inflammasome sensor recognizes the pathogen or threat signals and recruits the ASC adaptor protein, in turn recruiting the caspase 1 cysteine protease. Caspase 1 is synthesized as an inactive zymogen (45 kDa) that is activated via auto-cleavage to generate a heterodimer composed of 10-kDa and 20-kDa subunits serving as a molecular scissor to process pro-IL-1β and pro-IL-18. Caspase 1 also breaks up cells by generating holes in the membrane via the pore-forming protein GSDMD, resulting in inflammation-mediated pyroptosis [44,57].

## 4. Pathogenesis Triggered by the Inflammasome during COVID-19 Infection

Acute inflammation provides an efficient means to restore homeostasis following exposure to infection. However, inflammation can prove more harmful if the condition persists and underlies the pathogenesis of several diseases. Several investigations have recently concluded that SARS-CoV-2-induced hyper-inflammation is a primary cause of severe illness and death in COVID-19 patients. This hyper-inflammation is characterized by increased inflammatory markers [58] and pro-inflammatory cytokines [59] in patients’ serum. These inflammatory markers, such as C-reactive protein (CRP), serum ferritin, and procalcitonin, are associated with disease severity in COVID-19 patients [60]. Inflammasome activation can result in chronic inflammation in patients with COVID-19 (Figure 1), leading to severe disease conditions [61]. This severe condition is characterized by dysregulated cytokine release, pneumonia, and acute lung injury (ALI), which can rapidly progress to ARDS, disseminated intravascular coagulation, multisystem organ failure, and death.

### 4.1. Inflammation and Pulmonary Damage

Inflammation and lung damage are common symptoms in COVID-19 patients with both mild and severe symptoms. COVID-19 patients with other pulmonary diseases, such as chronic obstructive pulmonary disease (COPD) and other comorbidities, are at higher risk of severe outcomes [62]. Elevated serum levels of pro-inflammatory cytokines, such as IL-6, IL-8, and tumor necrosis factor α, are observed in immune cells of exacerbated cases of COVID-19. Hyper-inflammation in the lungs causes structural tissue damage such as alveolar destruction. Inflammasome development along the artery wall is considered as a feature of COVID-19-induced lung inflammation [63]. This alveolar damage will trigger the migration of other immune cells into the lungs [2]. Hyper-activation of the immune cells will cause the release of pro-inflammatory mediators and can damage different compartments of the lungs in severe COVID-19 cases. A multi-institutional autopsy cohort study revealed that diffuse alveolar damage (87% of cases), and large vessel thrombi (42% of cases) were observed in COVID-19 patients [64]. The presence of diffuse alveolar damage, severe capillary congestion, and various findings in the lungs and other organs suggests vascular dysfunction [65].

The mechanism of inflammasome activation in the lungs is accompanied by different pathophysiological processes. Infection of airway epithelial cells with SARS-CoV-2 can result in high levels of virus-linked pyroptosis and vascular leakage. Pyroptosis is a highly inflammatory form of programmed cell death that is frequently found in innate immune responses, mediated by inflammasomes [41,66]. Some DAMP molecules, such as extracellular high mobility group box 1 protein (HMGB1), are associated with inflammasome activation and severe lung damage [67]. Interferon induces the relocation of HMGB1 from the nucleus to the cytosol, and when there is an excess in the extracellular environment, it results in the release of pro-inflammatory cytokines IL-1 and IL-6. A high plasma level of HMGB1 is associated with ARDS in bacterial infections [68] and is considered a biomarker in severely ill COVID-19 patients. This protein promotes the recruitment of leukocytes to the lungs and causes free radicals, released by damaged cells, to contribute to inflammasome-induced lung damage [69].

In addition, other pulmonary diseases, including COPD, cystic fibrosis (CF), cigarette smoking (CS), and pulmonary fibrosis, are also associated with inflammasome activation and increased severity of disease in COVID-19 patients. SARS-CoV-2 can exacerbate asthma, another important risk factor for COVID-19-related morbidity [70]. Based on data obtained from previous COVID-19 cases, it is suggested that SARS-CoV-2 infection has severe implications in patients with pre-existing pulmonary fibrosis conditions [71]. NLRP3 components, such as IL-1β, drive upregulation of miR-155, which enhances the migration of fibroblasts [72]. According to a recent systematic review and meta-analysis study, both COPD and CS significantly contribute to the poor clinical outcome of COVID-19 patients [73]. Another meta-analysis study has also revealed similar outcomes [74]. COPD is associated with a fivefold increase in the development of severe COVID-19 infection because this phenomenon is related to impaired host immunity and microbiome dysregulation [74]. The dysregulation of the immune system associated with COPD in COVID-19 patients increases the risk of developing more severe symptoms [75]. 

### 4.2. Cardiovascular Damage

Cardiovascular comorbidities leading to high mortality rates have been reported in previous SARS outbreaks as well as in the current COVID-19 pandemic. The pooled prevalence of cardiovascular diseases in COVID-19 varies in different countries, ranging from 7.8% in China to 71.4% in Switzerland [76]. The ACE2 receptor provides the major entry point for SARS-CoV-2. The heart has a high expression of ACE2, paving the way for viral entry into cardiomyocytes and cardiac endothelial, mesenchymal, and immune cells [77,78]. The expression of inflammasome activation markers has shown correlation with that of cardiac markers, such as troponin, which provides evidence for inflammasome-mediated cardiovascular disorders in hospitalized COVID-19 patients [79]. Aberrant expression of pro-inflammatory cytokines in the heart following SARS-CoV-2 infection is involved in the onset and progression of cardiovascular diseases. These factors promote dysregulation of the cardiac ion channel, leading to cardiac arrhythmias, coagulation abnormalities, and subsequent heart failure [80]. Atherosclerosis is another common cardiovascular condition often linked with COVID-19 infection. Cholesterol crystals in atherosclerosis patients are DAMP molecules and are involved in NLRP3 inflammasome activation [81]. These cholesterol crystals can also induce complement-mediated inflammasome activation and the release of cytokines [82]. Endothelial cell damage in cardiovascular patients promotes pyroptosis and aggravates the inflammatory process [83]. Pyroptosis is defined by activating pathways that lead to the activation of NOD-like receptors, particularly the NLRP3 inflammasome and its downstream effector inflammatory cytokines [84]. The upregulation of inflammasome proteins, including NLRP3, caspase 1, IL-1β, and IL-18, is mediated by TXINP, a metabolic protein that regulates redox reaction and can act as a tumor suppressor [85]. Cytokine burst affects the stability of atherosclerotic plaques, eventually resulting in thrombosis and myocardial infarction [86]. The virus can also damage the heart indirectly via induction of hyper-inflammation in the lungs by the cytokine storm leading to myocardial infarction [87].

### 4.3. Neurological Damage

In many cases, COVID-19 patients present neurological symptoms such as encephalitis/meningitis, acute cerebrovascular disease, and Guillain–Barre syndrome (GBS) [88]. Patients who develop more severe COVID-19 symptoms are more likely to have brain-related complications. Reports have showed that 80% of hospitalized COVID-19 patients develop neurological manifestations [89]. Damage to the central nervous system is mainly attributable to the cytokine storm induced by COVID-19, similar to other respiratory viral infections [90]. Encephalitis is defined as acute inflammation of the brain mainly caused by viruses, whereas meningitis is inflammation of the brain and spinal cord membrane; both have been linked to SARS-CoV-2 infection [88]. Earlier cerebrospinal fluid analysis of a COVID-19 patient has revealed that the virus causes encephalitis not by directly invading the brain, but by inducing a cytokine storm [88]. Another study from Japan demonstrated an association of COVID-19 infection with the progression of viral meningitis [91]. This neurological damage has an indirect contribution to ARDS in COVID-19 patients besides the direct damage in the lungs [88].

An important role of inflammasomes in neurological complications has been reported in different settings. The main active component of oxidized low-density lipoproteins—lysophosphatidylcholine—plays an important role in cerebrovascular illness. It activates the NLRP3 inflammasome via G protein-coupled receptor 4 and causes apoptosis and inflammatory damage in brain microvascular endothelial cells [92]. Another study has found that Bruton’s tyrosine kinase, which physically interacts with ASC and NLRP3, is a critical component of the NLRP3 inflammasome. It contributes to ischemic brain injury by activating the inflammasome NLRP3 [93]. Owing to its role in the exacerbation of symptoms and tissue damage during viral infection, NLRP3 represents an attractive target for reducing the severity of COVID-19-associated neurological damage [40]. However, more research is needed to determine the precise mechanism of inflammasome activation in COVID-19 patients with neurological complications.

## 5. Potential Therapeutic Agents and Vaccine Strategies for COVID-19

COVID-19 has been declared by the World Health Organization (WHO) as a global health crisis because of its contagious nature and high mortality rates [1,94]. Urgent measures are required to stop the current spread of infection as well as the associated human and economic loses. At present, no effective therapeutic options are available for treating COVID-19 [95]. Despite this, the spectrum of medical therapies to treat COVID-19 is growing and evolving rapidly. COVID-19 is currently managed clinically with infection prevention and control measures as well as supportive care, including supplemental oxygen and mechanical ventilation support, as needed. The Food and Drug Administration (FDA) has approved remdesivir (Veklury) for the treatment of COVID-19 in hospitalized patients aged at least 12, and weighing at least 40 kg [96]. Other drugs, including dexamethasone, baricitinib, tofacitinib, tocilizumab, and sarilumab, are considered potential therapeutic options under specific circumstances [97,98]. Neutralizing monoclonal antibodies that bind to the SARS-CoV-2 S protein are alternative therapies given for non-severe COVID-19-infected patients. These neutralizing monoclonal antibodies remain active against the new delta variants of the virus [99]. Several gene-silencing approaches using RNAi and CRISPR technologies are also new targets for inhibiting SARS-CoV-2 and reducing the viral pathogenicity [100,101].

The recent development of a variety of RNA-based medications demonstrates the field’s enormous promises [102]. The first RNA-based therapy was approved by FDA to treat polyneuropathy in 2018 [103]. Furthermore, the increased efficacy seen in mRNA vaccines changes the view of the scientific world to look into RNA-based therapeutics for COVID-19. Researchers have discovered an RNA molecule that activates the early antiviral defense mechanism and protects mice from a variety of SARS-CoV-2 virus strains. Stem–loop RNA 14 (SLR14), a new family of RNA therapies, has been discovered. It is reported that a single dose of SLR14 reduced the mortality of K18-hACE2 mice after SARS-CoV-2 infection, which is auctioning through promoting interferon production and inhibiting viral load. It has also been shown that SLR14 provides better protection than other interferon I (IFN-I)–based antiviral therapies [104]. Further clinical studies are proposed to prove the safety and efficacy of the RNA-based therapeutic options for COVID-19 patients.

Host miRNAs represent an indigenous defense mechanism against a range of viral pathogens. MiRNAs are essentially small molecules involved in regulating the translation of mature messenger RNAs into proteins. These transcripts regulate the innate immune system and induce anti-viral effects by targeting the coding region or 3′ untranslated region (UTR) of the viral genome [105] (Table 1). Different host miRNAs have been shown to degrade viral mRNA and block its translation, as observed in influenza infection, thus inhibiting viral replication [105]. A recent study identified several miRNAs targeting ACE2 regulation in different tissue types. A few transcripts from the miR-200 family have been shown to regulate ACE2 expression and may have therapeutic efficacy in relieving COVID-19-related inflammation of the heart and other organ systems [106]. Sponging this transcript by heart-related circular RNA (HRCR; Table 1) can protect against adverse conditions such as cardiac hypertrophy and heart failure [107]. Both miR-181b and miR-146a are reported to protect the myocardium from atherosclerosis by regulating different components of the NF-ҡB pathway [108] (Table 1). The introduction of synthetic miRNA mimics for these two RNA types could provide cardioprotection during the onset of a COVID-19 infection. In addition, long non-coding RNAs (lncRNAs) have been proposed as a treatment option for cardiovascular diseases, such as atherosclerosis. Overexpression of antisense RNA in the INK4 locus (ANRIL) and myocardial infarction associated transcript (MIAT) lncRNAs in atherosclerotic plaques has been reported. On the other hand, the expression of the lncRNA metastasis-related lung adenocarcinoma transcript 1 (MALAT1) is lower in plaques [109]. The exploitation of the altered expression of lncRNAs provides new avenues for treating atherosclerotic inflammation during COVID-19 infection.

MiRNAs have also been suggested as treatment options for COVID-19 patients with neurological complications. For instance, miR-19b-3p modulates inflammation caused by the Japanese encephalitis virus and may therefore show utility in reducing brain inflammation in COVID-19 patients [115]. Acute cerebrospinal disease, especially in the form of ischemic stroke, is often observed in critical COVID-19 cases [88]. COVID-19-associated hypercoagulopathy and altered expression of ACE2 contribute to the etiology of ischemic stroke [117]. These miRNA therapeutics may provide a new hope to prevent the onset of stroke during COVID-19 [106]. GBS is another major neurological pathology of the peripheral nervous system attributable to an abnormal immune response caused by different viruses, including the coronavirus family [118]. A previous report indicates that miR-146a positively contributes to the pathogenesis of GBS by regulating inflammatory responses. Increased expression of miR-146a has been observed in GBS patients compared with controls; this finding is correlated with increased expression of IL-6 and CRP [116].

A recent in silico study identified 873 common miRNAs potentially targeting the COVID-19 viral genome [95]. The study further showed that the expression of some of these host miRNAs decreased with ageing, which may be an underlying reason for the lower incidence of infection in children compared with the elderly population [25]. To compensate for the decrease in indigenous miRNA in the elderly population, synthetic miRNA mimics [119] introduced via an effective delivery system could be useful for silencing of the COVID-19 genome. MiRNA antagomirs (AMOs) are synthetic miRNA antagonists that suppress the expression of complementary miRNA [120]. Demirci and colleagues recently predicted 29 potential SARS-CoV-2 precursor miRNAs that could target human genes [121]. AMOs may thus present an effective therapeutic strategy against viral miRNAs. In addition, AMOs may be effective against host miRNAs that facilitate replication of the viral genome in the host. AMOs against miR-146a could therefore present a potential therapeutic option for GBS [122]. Not only host miRNAs, but also SARS-CoV-2- derived miRNAs play a major role in regulating immune systems and metabolic pathways [123]. Inflammasomes play a dual role: protection against infections, versus exacerbation of symptoms during COVID-19 infection. Considerable evidence has shown that viral miRNAs regulate the expression of NLRP3 inflammasomes [124]. AMOs can also be utilized in such cases to fine-tune host inflammatory responses against SARS-CoV-2 infection.

Similarly, the rapid emergence of new SARS-CoV-2 variants demands the need for more innovative strategies that would help control the rapid spread of the virus. The CRISPR/Cas-based tools have been widely used for biomedical applications [125]. This technology provides an efficient therapeutic tool for preventing and controlling the spread of COVID-19 infection because certain orthologs of the Cas13 protein have been shown capable of suppressing viral and endogenous RNAs in mammalian cells [26,126]. Prophylactic antiviral CRISPR in human cells, a CRISPR/Cas13-based tool, was successfully used for the degradation of SARS-CoV-2 viral RNA in human lung epithelial cells. This strategy reportedly has the potential to inhibit more than 90% of coronaviruses, subject to the availability of an effective delivery system [26]. Another study used virus-free models and reprogrammed CRISPR/Cas13b tool targeting the transcripts derived from the S and N regions of the virus, achieving more than 98% silencing efficiency for the viral transcripts. They further demonstrated the inhibition of viral replication in infected mammalian cells following the action of Cas13b CRISPR RNAs. This strategy was found to be equally efficient in suppressing the mutant variants of SARS-CoV-2, hence providing an outstanding therapeutic tool for a wide range of current and emerging variants of SARS-CoV-2 [126].

For a pandemic like COVID-19, vaccines are now the only powerful weapons available to stop the rapid advance of the COVID-19 pandemic [127]. The current scenario is dynamic, and there are a number of significant unknowns. Limited vaccine supply, the introduction of novel viral variants, and vaccination-related complications are only a few of the issues that must be addressed [128,129]. Although much about the immune response to SARS-CoV-2 remains unknown, and vaccine-induced protective immunity may differ from natural immunity owing to the virus’s immune-evasion strategies, a better understanding of the natural immune response will aid in the development of effective vaccines and alternative therapeutic strategies [130]. Hence, COVID-19 vaccinations that are both safe and effective are the greatest strategy to avoid the pandemic’s continuation.

Several different types of vaccines are currently authorized for use or undergoing testing [27]. Currently, there are 114 vaccine candidates in clinical development and 185 vaccines in preclinical development [131]. Several platforms are being used to develop COVID-19 vaccines. Among the vaccines undergoing clinical evaluation, the lead vaccine candidates are viral-vectored and mRNA-based vaccines [130]. Other vaccine platforms include inactivated-virus vaccines and protein-subunit vaccines [132].

The mRNA vaccines, including BNT162 of BioNTech and mRNA-1273 from Moderna, are among the first candidates that were authorized for use [133]. The mRNA-based vaccines come with certain advantages over other vaccine types: they are easy to manufacture, and unlike protein-based vaccines, they do not require certain quality control tests. These vaccines work very well once released into the cytoplasm and are rapidly degraded after performing their job, since mRNA has a very short life [27]. Similar to other therapeutic molecules, mRNA vaccines also require a suitable therapeutic vehicle. Lipid-based nanoparticles (LNPs) are vehicles of choice because these vehicles ensure the safety and integrity of the mRNA during manufacturing and transportation [27]. Upon entry into the cytosol, the mRNA is translated into the S protein, and an immune response is triggered when the protein enters the circulation [127]. Studies suggest that such vaccines are highly effective and safe for individuals over 16 years of age and hence provide the only most effective tool for the battle against COVID-19 [134].

Inactivated vaccines are chemically modified SARS-CoV-2 viruses grown in cell culture and administered intramuscularly. The inactivated virus vaccines can induce a broad range of immune responses, including antibodies to different targets, such as M, N, and S proteins. However, such kinds of vaccine platforms have some limitations. These include the safety of growing live virus, possible disease outbreak resulting from inadequate inactivation, and questionable feasibility of providing an adequate vaccine for the global population [135].

SARS-CoV-2 proteins that are expressed in various systems, including insect cells, mammalian cells, and yeast cells, make up protein subunit vaccines. They do not require live viral replication, which makes production more manageable, yet yields are dependent on the variable capacity of spike protein to express. Recombinant spike protein and recombinant receptor-binding domain (RBD) vaccines are two protein subunit COVID-19 vaccines currently in development. The protein subunit vaccines target mainly the humoral immune response [132].

COVID-19 is characterized by hyper-inflammation and immunological dysfunction. Inflammasome activation causes hyper-inflammation, which contributes to cytokine storm-induced organ damage and mortality. COVID-19 vaccines have been developed to trigger the immune system against the SARS-CoV-2 virus and reduce morbidity and mortality. These vaccines work on a variety of platforms and can induce humoral and cellular immune responses [136]. The efficacy of vaccines is measured by the generation of long-lasting neutralizing antibodies [137]. However, there are no clear data on how long the antibodies last in the case of COVID-19 vaccines.

Messenger RNA-based vaccines showed higher efficacy (>95%) in protecting symptomatic COVID-19 infections when compared with other virus-based and protein-based vaccine platforms. These vaccine platforms enhance the production of Th1 cells and induce the production of neutralizing antibodies even after the first dose [138]. However, reduced efficacy of these vaccines has also been observed against the new variants of SARS-CoV-2 [139]. Not only adaptive immune response, but also vaccines, may improve innate immune response by inducing a type I interferon (IFN-I) response. The mRNA vaccines use pure, in vitro transcribed single-stranded mRNA with modified nucleotides to minimize binding to TLR and immunological sensors—decreasing excess IFN-I generation and its negative effect on cellular translation [140]. Increased IFN-I generation is associated with cellular damage and immune dysfunction in severely ill COVID-19 patients [141]. IFN-I has been shown to function as an antiviral defense and reduce viral progression at the early phase of SARS-CoV-2 infection [142]. In this regard, COVID-19 vaccines may play a significant role in reducing immune dysregulation and inflammasome activation. The possible mechanisms include the following: (1) the vaccine inhibits the viral replication so as to decrease the activation of inflammasomes in response to SARS-CoV-2 and its derivatives, which function as PAMPs [54,55,56]; (2) the vaccine triggers the immune system and hence reduces ultimate intracellular damage and cell death. The reduced cell damage and cell death are associated with the suppression of the release of endogenous DAMPs, including Ca^2+^ and ROS. This prevents the activation of inflammasome sensor molecules, halts the release of pro-inflammatory markers, and prevents hyper-inflammation. However, further evidence is needed to understand the potential role of the different vaccine platforms in reducing COVID-19-associated inflammasome activation.

NcRNAs, including miRNAs and their synthetic counterparts, provide novel avenues for the treatment of COVID-19. Further in-depth studies are required to establish the pros and cons of each therapeutic approach for efficient utilization in clinical settings.

## 6. Non-Coding RNAs: Potential Therapeutics for Inflammasome-Induced COVID-19 Pathogenesis

Based on their length, ncRNAs are classified into short ncRNAs (<200 nucleotides) and lncRNAs (>200 nucleotides) [143]. Owing to their functions in disease pathogenesis, ncRNAs, also known as non-protein-coding genomes, have attracted considerable interest in biology and medicine. While several types of ncRNAs play critical roles in cellular homeostasis and disease, miRNAs are the most extensively studied [144].

**Figure 2:**
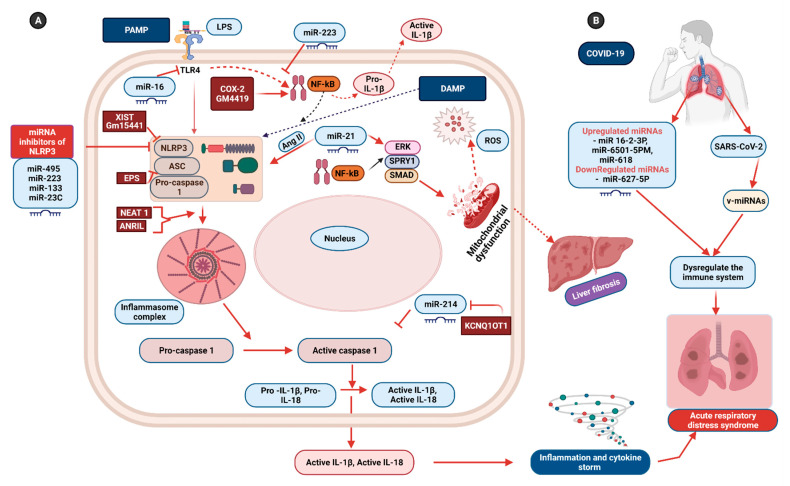
The mechanism of NLRP3 pathway activation and its regulation by ncRNAs. (**A**) The priming of NLRP3 occurs when TLR4 senses some PAMPs and activates the NF-kB pathway, which helps in the expression and release of pro-IL-1β. Both PAMP and DAMP molecules result in the inflammasome activation and release of pro-inflammatory cytokines. Non-coding RNAs can regulate the expression of NLRP3 inflammasome at various stages. miR-16 blocks NLRP3 activation by specifically targeting TLR4 receptors. MiR-495 and miR-223 specifically target the NLRP3. MiR-214 binds caspase 1. The lncRNA EPS inhibits the expression of ASC protein. ncRNAs also positively regulate the activation of NLRP3 inflammasome. MiR-21 regulates NLRP3 expression via ERK/SPRY/SMAD pathways, and results in mitochondrial dysfunction and ROS generation, which trigger inflammasome activation. This process induces liver fibrosis. The lncRNA NEAT1 helps in the assembly of NLRP3 and downstream factors, such as caspase 1 and IL-1β. KCNQ1OT1 binds miR-214, thus preventing its binding with caspase 1. The lncRNA COX-2 and GM4419 enhance NLRP3 expression by interacting with NF-kB. The lncRNA ANRIL regulates NLRP3 by the upregulation of the BRCC3 gene. The lnc Gm15441 and lnc XIST suppress the expression of NLRP3. (**B**) miRNAs in patients with COVID-19 and SARS-CoV-2-derived miRNAs dysregulate the immune system, resulting in ARDS. Ang II—angiotensin II; ANRIL—antisense RNA in the INK4 locus; ARDS—acute respiratory distress syndrome; ASC—apoptosis-associated speck-like protein containing a caspase activation and recruitment domain; COVID-19—coronavirus disease 2019; COX-2—Cyclooxygenase-2; DAMPs—damage-associated molecular patterns; EPS—epidermal growth factor receptor pathway substrate; ERK—extracellular regulated MAP kinase; IL-1β—interleukin 1 beta; LPS—lipopolysaccharide; lncRNA—long non-coding RNA; miRNA—microRNA; NEAT 1—nuclear paraspeckle assembly transcript 1; NF-kB—nuclear factor kappa light chain stimulation of activated B cells; NLRP3—nucleotide-binding oligomerization domain, leucine-rich repeat, and pyrin domain-containing 3 protein; PAMPs—pathogen-associated molecular patterns; ROS—reactive oxygen specious, SARS-CoV-2—severe acute respiratory syndrome coronavirus 2; SMAD—mothers against DPP homolog 2; SPRY1—sprouty RTK signaling antagonist 1; TLR—Toll-like receptor; v-miRNAs—viral microRNAs; XIST—X-inactive specific transcript. The symbol “⊥” indicates inhibition; solid arrow indicates the direction of effect; dotted arrow indicates alternative mechanisms.

A recent study provides significant insights into the function of ncRNAs, including miRNA, in the fine control of NLRP3 inflammasomes in both nuclear and cytoplasmic compartments via different mechanisms [10] (Figure 2). These miRNAs represent an efficient tool for regulating the NLRP3 complex in diverse inflammatory disorders. Ning and colleagues have recently demonstrated that miR-21 regulates activation of NLRP3 through SPRY1/ERK/NF-ҡB pathways in liver tissue [145]. According to their study, miR-21 targets SPRY1 and Smad7 to mediate angiotensin-II-induced NLRP3 inflammasome activation in primary hepatic stellate cells. Overexpression of miR-21 is reported to increase the intracellular oxide content and promote mitochondrial dysfunction [145]. MiR-21, which is significantly expressed in the lungs of patients with idiopathic pulmonary fibrosis, may also regulate NLRP3 in the case of pulmonary fibrosis [146].

A similar study has demonstrated that overexpression of miR-495 inhibits activation of NLRP3 inflammasome and prevents progression to ALI, caused by subsequent inhibition of inflammasome-induced alveolar inflammation and pyroptosis, while methylation of the miR-495 promoter promotes progression into ALI (Figure 2) [147]. MiR-223 additionally shows utility in reducing inflammation and preventing the onset of ALI and ARDS by suppressing NLRP3 as well as Toll-like receptor 4 (TLR4)/NF-ҡB pathways in the in vitro model of ALI [148]. MiR-223 specifically targets a highly conserved site in the 3′ UTR region, restricting activation of NLRP3 inflammasome [124]. In addition to miR-495 and miR-223, miR-16 also inhibits the expression of NLRP3 during ALI by directly targeting TLR4 [149]. TLR4 belongs to PRRs that recognize PAMPs such as lipopolysaccharides. A critical role of miR-181a-2-3p in regulating the inflammasome during pulmonary disorders, such as COPD, has also been reported. The group found that the silencing of the transcript resulted in the activation of inflammasomes along with overexpression of TLR4 and sequestosome-1 genes [150].

Inflammasome activation is important for host defense against viral infections, because these molecular sensors can trigger an innate immune response against invading pathogens. Emerging evidence has shown how different viruses regulate inflammasome activity via miRNAs. One such example is Epstein–Barr virus (EBV) miR-BART15 targeting the miR-223 binding site in NLRP3 3’ UTR site. Secretion of this miRNA from EBV-infected B cells was observed using exosomes as vehicles for delivery into non-infected cells [124]. NLRP3 clearly plays a critical role during hepatitis virus infection and promotes hepatocellular carcinoma. Overexpression of miR-223 in Hep3 B cells resulted in the suppression of cell proliferation along with apoptosis. These changes were associated with lower activity of NLRP3 and its downstream factors (caspase 1, IL-1β and IL-18) following miR-223 overexpression in vitro [151].

Like miRNAs, lncRNA can maintain the homeostasis of the immune system by preventing dysregulation of the NLRP3 inflammasome. These transcripts regulate NLRP3 in both positive and negative manners under various cellular conditions [10] (Figure 2). The lncRNA nuclear enriched abundant transcript 1 (NEAT 1) has been shown to regulate NLRP3 in a positive manner, facilitating assembly of the complex as well as maturation and secretion of its downstream factors—caspase 1 and IL-1β. Furthermore, in a murine model of pneumonia and peritonitis, deficiency of the NEAT 1 transcript significantly reduced inflammatory responses [13]. The lncRNA MALAT-1 also promotes expression of the NLRP3 inflammasome by competing with miRNAs, such as miR-133 and miR-23C, which potentially inhibit expression of the NLRP3 complex [14,152]. Maternally expressed gene 3 (MEG-3) is another lncRNA transcript that influences the regulation of the NLRP3 inflammasome by neutralizing the inhibitory effects of miR-223 on inflammasome activation [153]. The lncRNA ANRIL also positively influences the regulation of the NLRP3 inflammasome. The pathogenic effects of ANRIL were determined in a study showing its activity as a sponge for miR-122-5p, facilitating activation of NLRP3 via upregulation of BRCC3 gene expression [154].

The lncRNA KCNQ1OT11 targets and binds miR-214, and hence prevents the binding of miR-214 to caspase 1, while miR-214 is reported to confer an inhibitory effect on caspase 1 activity (Figure 2). In brief, KCNQ1OT1 triggers pyroptosis via the miR-214/caspase 1 pathway [155]. The lncRNA COX-2 promotes the expression of genes associated with NLRP3. The inflammatory state is initiated by COX-2-assisted nuclear translocation of NF-ҡB p65, and deficiency of the COX-2 transcript results in alleviation of NLRP3 expression along with the reduced secretion of IL-1β [15]. Gm4419 is another key lncRNA involved in the activation of inflammation in certain pathologies. Gm4419 activates NF-ҡB by directly interacting with its p50 subunit and further activating the NLRP3 complex, and the activation of NF-α results in the onset of inflammation, fibrosis, and other adverse phenomena [156].

A number of lncRNAs also modulate the immune response by negatively regulating the NLRP3 inflammasome or its components (Figure 2). For example, the lncRNA EPS suppresses the activation of NLRP3 by inhibiting ASC protein [16], while the lncRNA Gm15441 suppresses NLRP3 by inhibiting its own antisense transcript TXINP, which functions in NLRP3 activation and downstream inflammation. Upregulation of Gm15441 is stimulated by metabolic stress-induced activation of the nuclear receptor peroxisome proliferator-activated receptor-alpha [17]. The lncRNA X-inactive specific transcript (XIST) has also been identified as a negative regulator of the NLRP3 complex. An earlier study has shown that silencing of XIST is associated with enhanced expression of NLRP3 components. However, inhibition of NF-ҡB results in reduced XIST activation. Accordingly, it is proposed that the NF-ҡB cascade promotes the expression of XIST while XIST controls the process of inflammation by negatively regulating the NLRP3 pathway [18].

Recent clinical and computational studies on COVID-19 patients reported the presence of miRNAs and predicted their role in the disease pathophysiology. The prediction showed that both virus and host miRNAs might play essential roles in the pathogenesis of COVID-19. Upregulation of miRNAs, such as miRNA-16-2-3p, miRNA-6501-5p, and miRNA-618, and downregulation of miRNA-627-5p in peripheral blood, were reported in COVID-19 patients [157]. SARS-CoV-2 uses its RNA genome as a sponge to adsorb host functional miRNA and disrupt the immune system, providing an opportunity for SARS-CoV-2 to kill host cells [158]. This may help the virus by promoting its survival in the infected cells and thus continuity of its replication cycle.

In addition to host miRNAs, studies also showed the presence of SARS-CoV-2-derived miRNAs. Recently our research group have demonstrated how miRNAs derived from the SARS-CoV-2 genome regulate host metabolic pathways [123]. In this study, SARS-CoV-2 v-miRNAs were identified by deep sequencing in infected Calu-3 and Vero E6 cell lines. Accordingly, ten v-miRNAs were identified in the *N* gene along with 5′ end of the viral genome (*5′UTR*), *orf1ab*, *orf2* (Spike; S), *orf5* (Membrane; M), *orf7a*, and *orf10* of SARS-CoV-2, which mainly include the coding regions for structural and accessory proteins [159]. We have also shown that v-miRNAs derived from the N gene are differentially expressed in COVID-19 patients [123].

These microRNAs and lncRNAs could be used to target specific SARS-CoV-2 motifs. According to a study, the SARS-CoV-2 genome contains motif sequences in the 5′ UTR leader sequence that can be selectively recognized by specific human miRNAs and lncRNAs, such as H19, Hotair, Fendrr, and LINC05 [160]. Our study also demonstrated that SARS-CoV-2 derived v-miRNA-N-28612 could directly bind with inflammasome genes *CASP 1* and *IL-1β* [123]. Additional studies are required to establish the link between SAR-CoV-2 v-miRNAs and the regulation of NLRP3 inflammasome. NLRP3 plays a dual role in protecting against invading pathogens as well as triggering the cytokine storm that can lead to the development of severe symptoms during viral infection. Therefore, miRNAs could be effectively applied as a fine regulator for the sustained activity of NLRP3 during COVID-19 infection. As a result, it is essential to comprehend the mechanism and create miRNA interferences that target these miRNAs.

## 7. Delivery Strategies for Synthetic RNA-Based Therapeutic Molecules

**Figure 3:**
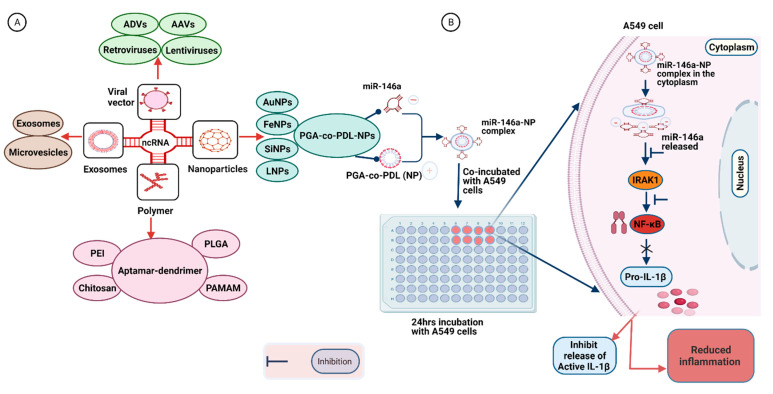
Vectors currently in use for the delivery of therapeutic ncRNAs. (**A**) Shows the different vectors and their subcategories, include viral vectors, polymers and their derivatives, nanoparticles and their derivatives, and exosomes-based delivery systems. (**B**) Shows the delivery of miR-146a in to A549 cells. Negatively charged miR-146a is adsorbed with a positively charged PGA-co-PDL (NPs) when co-incubated with A549 cells. After 24 h of incubation, the miR-146a-NPs complex enters into the A549 cells and is released into the cytoplasm. The released miR-146a then inhibits the expression of the *IRAK1* gene, thus stopping the induction of pro-inflammatory cytokines via the NF-ҡB pathway and resulting in reduced inflammation. A549 cells—adenocarcinomic human alveolar basal epithelial cells; AAVs—adeno-associated viruses; ADVs—adenoviral vectors; AuNPs—gold nanoparticles; FeNPs—iron nanoparticles; IL-1β—interleukin 1 beta; IRAK1—interleukin-1 receptor-associated kinase 1; LNPs—lipid-based nanoparticles; miR-146a—microRNA 146a; NF-kB—nuclear factor kappa light chain enhancer of activated B cells; PAMAM—poly (amidoamine); PEI—polyethyleneimine; PGA-co-PDL-NPs—poly(glycerol adipate-co-ω-pentadecalactone nanoparticles; PLGA—poly-lactic-co-glycolic acid; SiNPs—silicon nanoparticles. The symbol “⊥” indicates inhibition; solid arrow indicates the direction of effect, the symbol “−”indicates negative charge; the symbol “+” indicates positive charge.

NcRNAs present a novel therapeutic target for respiratory diseases and viral infections. However, several challenges need to be addressed for the effective utilization of ncRNAs as a potent therapeutic tool [161]. These transcripts cannot survive for long periods of time in blood as they are rapidly cleaved by nuclease enzymes [162]. Moreover, transcripts are unable to penetrate the cell membrane, owing to their molecular weights and polyanionic nature [163]. The therapeutic efficacy of naked ncRNAs is further reduced by their non-specific activity, potentially leading to adverse side effects and unwanted phenotypes [163]. NcRNAs also trigger the innate immune response in the host tissue, leading to unintended immunotoxicity [164]. Therefore, an efficient delivery system that can precisely deliver RNA cargos and their mimics to target organs or cells remains an urgent unmet medical need. To efficiently deliver miRNAs for therapeutic purposes, both viral and non-viral miRNA delivery mechanisms are currently in use, each with its own benefits and drawbacks (Figure 3 and Table 2).

New concepts in human therapeutics are emerging as a result of advances in our understanding of RNA biological processing and regulation with functional implications in the therapies for respiratory viral infections [165]. Various delivery systems have been designed for the targeted delivery of therapeutic RNA and other therapeutic agents for various diseases. These delivery systems are tested usually with small animals (mouse or rat) and sometimes with cell lines.

### 7.1. Virus-Based Vectors

The application of RNA viruses to transiently deliver genetic information for cell manipulation in basic research and clinical therapy is an increasingly common practice. Multiple viral vectors have been employed for gene transfer into a particular target site. Viral vectors are viruses that have been modified for the purpose of gene delivery, including adenoviruses, adeno-associated viruses (AAVs), and retroviruses. These viruses lack replication properties but can ensure targeted delivery and constant expression of their cargo [189,190].

Adenoviral vectors (ADV) are the most commonly used viral vectors for clinical trials of gene therapeutic agents. ADVs have an icosahedral protein capsid and a 36-kb linear duplex DNA genome that can carry around 8–30 kb foreign cargo with the ability to transfect both dividing and non-dividing cells with high expression levels [191,192]. ADVs are used for the in vivo delivery of short hairpin RNA (shRNA) into tumor cells, where they have proven effective in inhibiting tumor growth and angiogenesis [166]. The most widely used ADVs for gene therapy are first-generation vectors, which are depleted of E1 with or without E3 knockout. The disadvantages of using ADVs include poor vector stability with small RNA cassettes, the requirement for repeated administration, triggering of the immune response, and toxicity to host tissue in some cases [192].

AAVs can also deliver small RNA payloads, owing to their small ssDNA genome containing only two genes that can be replaced with foreign genetic material, and require adenoviruses for replication and vector production. However, engineered AAVs can perform these functions on their own [193,194]. AAV vectors can efficiently deliver RNA to their target cells or tissues with low immunological host responses. These vectors are widely used for the delivery of shRNA both to cell lines and animal models, and have shown promising results in enhancing the therapeutic efficacy of their cargo [195].

Retroviruses represent another class of vectors that perfectly integrate their genomes into the host, ensuring the long-term expression of the delivered payload. The viral genome comprises a minimum of three genes and can be replaced with the gene of interest because viral entry into the host is independent of viral proteins [192]. To date, different retroviral vectors have been used to introduce small interfering RNA (siRNA) into cells for cancer therapy [196]. Lentiviruses represent a subclass of retroviruses with an ssRNA genome that can accommodate a large-sized payload. They are even capable of transducing non-dividing cells such as neurons [197]. These vectors are widely used for the transfer of RNA of interest into target regions, such as local delivery into brain cells via injection [167]. While viral vectors represent an effective option as a delivery vehicle, their utility is limited by a few factors, such as lack of large-scale production facilities, immunogenicity, and toxicity.

### 7.2. Polymer-Based Vehicles

Biodegradable polymers are more widely used as a delivery system for miRNAs than viral vectors, owing to their low toxicity and versatile chemical nature [189]. Polyethyleneimine (PEI) is among the early polymers commercially employed for targeted gene delivery because of its high nucleic acid binding and transfection capabilities. This polymer possesses a high buffering capacity that can deactivate lysosomal nuclease enzymes, thus protecting the integrity of its nucleic acid cargo [168]. Poly-lactic-co-glycolic acid (PLGA) is the most widely accepted FDA-approved vehicle for delivering drugs and nucleic acids, including ncRNAs [198]. PLGA is a copolymer of poly(lactic acid) and poly(glycolic acid), and the rate of biodegradability largely depends on the ratio of the two polymers. Degraded products are usually removed via normal cellular metabolic pathways [198,199]. However, due to its hydrophobic property, PLGA is less effective for miRNA delivery. PLGA can be modified with other molecules, such as polyethylene glycol (PEG), lactobionic acid, and vascular endothelial growth factor antibody. Such modified PLGA is reported to serve as an efficient tool for the delivery of therapeutic miRNA cargo [171]. For instance, in an earlier study, siRNA cargo was condensed with PEI, PEG, and PEG–PEI polymers for a gene knockout study in the lungs of mice. While no significant histological abnormalities were identified, siRNA condensed with PEG–PEI was found to be more effective while showing pro-inflammatory potential.

On the other hand, the RNA payload conjugated with PEI only was separated in the lungs and rapidly excreted [169]. Poly(ester amine)-alt-PEG is another PEI-derived polymer that can effectively deliver therapeutic agents to the respiratory system. SiRNA targeting the Akt1 transcript in the lungs was aerosolized with the polymer and suppressed lung cancer progression in a mouse model with no side effects [174]. Polyurethane-short branch-polyethylenimine (PU-PEI), utilized as a vehicle for delivery of miR-145 into a population of cancer stem cells of lung adenocarcinoma, as well as xenograft tumors, inhibited epithelial-mesenchymal transdifferentiation (EMT) and prevented tumor growth [170]. MiRNA nanomedicines were prepared for the treatment of CF by complexing miR-126 with either PEI or chitosan polymers. The PEI-based strategy was more effective in knocking down target gene in the cell model of CF [112]. In another study, human alveolar adenocarcinoma A549 cells were incubated with miR-146a adsorbed onto PGA-co-PDL NPs for 24 h [175] (Figure 3). This strategy resulted in attenuated expression of IRAK1, which plays a role in the induction of pro-inflammatory cytokines via the NF-ҡB pathway [200].

Chitosan is another natural polymer with diverse biological properties and can effectively deliver ncRNAs or their mimics into host cells [41]. Chitosan and its derivatives are widely used for the delivery of miRNAs, owing to their positive charges that can form multilayered complexes with negatively charged transcripts. However, their biological and physiochemical properties may be affected by several factors, such as the ratio of chitosan to nucleic acid and molecular weight [172]. Dendrimers are large, branched polymers that interact with different molecules via covalent conjugation, encapsulation, and electrostatic interactions. Polyamidoamine (PAMAM) is among the most widely studied dendrimers and has been shown to be an effective vehicle for targeted delivery of miRNAs upon modification with an aptamer [173,201].

### 7.3. Nanoparticle-Based Vectors

Nanomaterials are at the forefront of the fast-paced growth of nanotechnology. These materials are superior and indispensable in several human activities due to their unique size-dependent properties. An NP, also known as an ultrafine particle, is a small particle with a diameter of 1 to 100 nm. NPs also provide a valuable option for the controlled delivery of ncRNAs for therapeutic purposes. These particles facilitate targeted and protected delivery of their payloads, thus showing enhanced therapeutic potential [202]. ANP-based self-assembling formulation, comprising a cyclodextrin-containing polymer, PEG, a human transferrin ligand, and a transferrin receptor, was utilized for the intravenous delivery of siRNA into patients in a clinical trial setup [21]. NPs derived from hyaluronic acid (HA) are used as carriers of various biological and pharmacological agents. Previous studies have shown that HA-coated PEI-PLGA-derived NPs can efficiently deliver miRNA along with a chemotherapy drug [107].

LNPs are also commonly used delivery systems, since lipid is the main component of the cell membrane. Interactions of lipid with the cell membrane facilitate uptake of the RNA payload by target cells [203]. The strong electrostatic interactions between positively charged lipid and negatively charged RNA molecules protect the latter from enzymatic degradation and promote attachment to the cell membrane [203]. However, the lipid molecule can also confer cytotoxicity by damaging the cell membrane, vacuolizing the cytoplasm, and forming aggregates with negatively charged serum proteins. Therefore, neutral lipids or other molecules, such as PEG, are often applied to reduce the toxicity of LNPs and increase their therapeutic potency [177,204,205]. LNPs prepared from inorganic materials are also used for a variety of applications in nanomedicine. Iron-based nanomaterials can efficiently deliver miRNAs with a high level of cellular uptake [179]. Silica-based nanostructures are also highly stable and biocompatible delivery molecules. MiRNA conjugated to mesoporous silica NPs effectively achieved therapeutic goals in a previous clinical study [180]. Gold nanoparticles (AuNPs) are mainly enhanced with specific functional groups to achieve optimal miRNA entrapment and cellular uptake [181].

### 7.4. Exosome-Based Vectors

Exosome-based carriers have emerged as a new class of vehicles that exhibit high safety and bioavailability along with low toxicity and immunogenicity. Exosomes represent a class of endogenous NPs secreted by many different cell types. These molecules are characterized by size, chemical composition, and the ability to carry various cargo types (such as RNA and proteins) and are involved in regulating intracellular communications. Alterations in exosome functions are associated with several disorders and are of significant value as diagnostic and therapeutic tools [206]. Exosomes serve as robust carriers for RNA-based drugs.

Bryniarski and colleagues successfully demonstrated T-cell tolerance in allergic cutaneous contact hypersensitivity mice through miR-150 transfer by exosome-like nanovesicles regulating T-cells [183]. In another study, miR-150 packaged into THP-1 cell-derived microvesicles enhanced endothelial cell migration in human HMEC-1 cells [188]. Connective tissue growth factor drives the fibrosis of hepatic stellate cells. Exosomal delivery of miR-214 downregulated this growth factor, thus blocking the fibrogenesis pathways in hepatic cells [184]. An anti-miR-9 therapeutic was delivered via MSC-derived exosomes to glioblastoma multiform cells. This treatment resulted in the downregulation of miR-9 and lower resistance to the chemotherapeutic drug temozolomide [185]. Shuttling of siRNA via exosomes in both human and mouse hepatic cells enhanced the therapeutic efficiency of RNAi-based drugs in viral hepatitis along with other liver diseases [187].

These studies clearly support the potential of exosomes as a reliable vehicle for the delivery of RNA-based therapeutics. However, their use is limited by the lack of an efficient method to load RNA cargo into the exosome [207]. Specific parameters, such as incubation time, temperature, volume and RNA-to-extracellular vesicle ratio, are crucial for the efficient delivery of RNA-based therapeutics to target sites. Further optimization of these parameters is required to accelerate the use of exosomes as carriers for ncRNAs and other drugs [208]. In addition to exosomes, exosome-mimetic nanovesicles may also be employed for targeted drug delivery. While these molecules have similar properties, their production yield is 100 times more than that of exosomes [209]. Similarly, the engineering of exosomes for delivering different drugs presents an effective way to treat different respiratory diseases. In one such study, miR-126 loaded into exosome (231-exo) successfully suppressed the proliferation and migration of A549 lung cancer cells [186].

Given mounting evidence that the most harmful SARS-CoV-2 reactions occur predominantly in the respiratory system, locally targeted therapy delivery via proper delivery vehicles is critical. Experiments are currently underway to test various delivery vehicles for vaccine development and other treatments. Viral vectors or virus-like particles are being used in a number of ongoing COVID-19 vaccine candidate trials [210]. Adenoviruses are by far the most widely used and technologically advanced viral vectors for SARS-CoV-2 vaccinations [211]. Although more data are needed on COVID-19, understanding various delivery mechanisms will aid in the development of viable treatments and vaccines against SARS-CoV-2. Further, more evidence is needed to fully comprehend the advantages and disadvantages of the various delivery vehicles for SARS-CoV-2 vaccines and treatments.

## 8. Conclusions

The COVID-19 pandemic has emerged as the worst health crisis the modern world has ever witnessed, causing substantial human and economic losses. The disease’s severity ranges from mild pneumonia cases to life-threatening conditions such as ARDS. SARS-CoV-2, along with its different variants, can activate the NLRP3 inflammasome either via altering the expression of ACE2 protein or the production of ROS and ionic imbalance in the cell membrane. NLRP3 inflammasome activation could trigger a cytokine storm in the lungs as well as other organs, ultimately leading to organ failure and death. Regulation of inflammasome is one avenue that could significantly reduce COVID-19-inflicted damage. NcRNAs and their synthetic counterparts represent one of the most efficient tools to regulate inflammasomes at different levels. These transcripts act via different mechanisms and could relieve severe symptoms, with potential to save human lives if used as therapeutic agents. However, the utility of ncRNAs is limited, owing to various challenges, such as short half-life, low stability in fluids, immune reaction, and unintended toxicity. The lack of an efficient delivery system also limits their use as a potent and safe therapeutic option. Recently, exosome-derived carriers have been investigated as a new type of vector for delivering biological cargo into target sites. These carriers are less toxic because they are secreted by different types of cells in the body, but their utility as a biological vector is yet to be established. Further research is warranted to fine-tune these vectors for optimizing their therapeutic effects. In conclusion, considering ncRNAs, coupled with efficient and safe delivery systems, should provide an effective targeted therapeutic option for managing COVID-19-associated immune dysregulation and future global health challenges.

## Figures and Tables

**Table 1:** The therapeutic potential of ncRNAs in different diseases and the mechanisms involved.

Potential Therapeutic Ncrna	Disease	Potential Mechanisms
LNCRNAS ^1^		
SHRNA-TUG1	COPD	Preventing airway remodeling and inflammation by the knockdown of the lncRNA TUG1 [110]
SHRNA-PVT1	Asthma	Regulation of inflammation by the knockdown of lncRNA PVT1 [111]
SHRNA-XIST, SHRNA-TLR8-AS1	Cystic fibrosis	Preventing inflammation by downregulating lncRNAs XIST and TLR8-AS1 [112]
HEART-RELATED CIRCULAR RNA (HRCR)	Myocardial infarction	Sponge the pro-inflammatory effects of miR-223 [87,107]
MIRNAS ^2^		
ANTI-MIR-21	Asthma	Regulation of inflammation by the knockdown of miR-21 [113]
ANTI-MIR-126	Cystic fibrosis	Regulation of innate immune response [114]
ANTI-MIR-155	Pulmonary fibrosis	Downregulation of NLRP3 inflammasome by silencing miR-155 [72]
MIR-200 FAMILY	Cardiac inflammation	Knockdown of ACE2 [106]
SYNTHETIC MIRNA MIMICS OF MIR-181B AND MIR-146A	Atherosclerosis	Prevent inflammation [108]
SHRNA	Atherosclerosis	Targeting lncRNA ANRIL and MIAT [109]
MIR-19B-3P	Encephalitis	Reduction in inflammation [115]
MIR-200	Ischemic stroke	Knockdown of ACE2 [106]
ANTI-MIR-146A	GBS	Downregulation of inflammatory response [116]

^1^ long non-coding RNAs; ^2^ microRNAs.

**Table 2:** Different vectors for the delivery of ncRNAs, their therapeutic effects and potential limitations.

Delivery Vector	Payload	Target	Therapeutic Impact	Limitations
Viral Vectors				
Adenovirus vectors/Adeno-associated viruses	ShRNA (sh-VEGF), ShRNA (sh-Hec1)	Endothelial cells, SF9 tumor cell line	Inhibits tumor growth and angiogenesis, depletion ofHEC1 protein in SF9 cells	Poor vector stability, trigger immune response, cytotoxicity [166]
Lentivirus vectors	ShRNA	Cortical neurons	Target gene knock down	Not reported [167]
Non Viral Vectors				
PEI/PEG–PEI polymer/PU-PEI	MiRNA mimics, DsiRNA, MiR-145	Lungs	Elevates pulmonary miRNA levels, knock-down of target genes, inhibits EMT and tumor growth	Lacks pulmonary selectivity, moderate inflammatory effects [168,169,170]
PLGA	MiR-99a	Hepatic carcinoma	Downregulation of target genes, reduction in tumor size	Not reported [171]
Chitosan	MiR-145	MCF-7 breast cancer cells	Downregulation of target mRNA	Not reported [172]
PAMAM	MiRNA	Prostate cancer (PCa) cells/xenograft mouse model	Enhanced survival of tumor-bearing mouse	Not reported [173]
Poly (ester amine)-alt-PEG	SiRNA	Lungs	Suppressed progression of lung cancer	Not reported [174]
PEI/Chitosan	MiR-126	Cystic fibrosis (CF)	Knockdown of target gene	Not reported [112]
PGA-co-PDL	MiR-146a	COPD	Reduced expression of the *IRAK1* gene	Not reported [175]
Aptamer-dendrimer	MiR-34a	Lung cancer	Reduced cancer cell growth, invasion, induced apoptosis	Not reported [176]
Hyaluronic acid coated PEI-PLGA NPs	MiR-542-3p and doxorubicin	Breast cancer	Tumor cell apoptosis	Not reported [107]
Lipid-based nanoparticles	MiR-122 mimics, miR-145	Hepatocellular carcinoma, lungs	Suppression of target genes and tumor xenograft, reduced pulmonary hypertension	Low cytotoxicity [177,178]
Iron-based nanoparticles	MiR-let7a	Brain cancer cells	Enhances apoptosis of cancer cells	Not reported [179]
Silica-based nanostructures	Anti-miR-221	Glioma cells	Induction of apoptosis	Not reported [180]
Gold nanoparticles	MiRNA mimics, SiRNA	Cancer cell lines, lung cancer	Affected proliferation and target gene expression, reduced cancer cell proliferation and tumor growth	Not reported [181,182]
Exosomes	MiR-150, miR-214, anti-miR-9, miR-126, siRNA	Allergic cutaneous sensitivity (mouse), hepatic cells, lung cancer, glioblastoma cells	T-cell regulation, halting of fibrosis, downregulation of MiR-9, supressed the migration and proliferation of cancer cells, enhanced therapeutic efficiency of RNAi drug	Not reported [183,184,185,186,187]
THP-1 cell-derived microvesicles	MiR-150	HMEC-1 cells	Enhanced endothelial cell migration	Not reported [188]

## Data Availability

Not applicable.
